# AlSi10Mg Heat Sinks for Passive Cooling: Convective–Radiative Performance of Finned and Triply Periodic Minimal Surface Designs via Laser Powder Bed Fusion

**DOI:** 10.3390/ma19173709

**Published:** 2026-08-31

**Authors:** Josef Thomas Hollaman, Adil Saeed, Zulfiqar Ahmad Khan, Thomas Singleton

**Affiliations:** 1 NanoCorr, Energy and Modelling Research Group, Bournemouth University, Poole BH12 5BB, UK; 2 Department of Mechanical Engineering, New Uzbekistan University, Tashkent 100007, Uzbekistan; 3 State Key Laboratory of Tribology in Advanced Equipment, Tsinghua University, Beijing 100084, China

**Keywords:** AlSi10Mg, lattice structures, gyroid lattice, additive manufacturing, direct metal laser sintering, natural convection, radiative heat transfer, thermal resistance, passive heat sink, electronics cooling

## Abstract

Increasing power densities in electronic systems require efficient passive thermal-management solutions. This study numerically compares five DMLS-compatible AlSi10Mg heat-sink architectures: perforated planar-fin, curved radial-fin, Schwarz P, BCC diamond and gyroid designs. Conjugate heat-transfer simulations incorporating conduction, natural convection and surface radiation were performed under an identical 18 W thermal load. Performance was evaluated using surface temperature, thermal resistance, temperature uniformity and convective–radiative heat dissipation. The gyroid achieved the lowest average surface temperature (82.02 °C) and combined thermal resistance (3.30 °C W^−1^), whereas the BCC diamond provided the greatest temperature uniformity (UI = 0.035). Radiation contributed 46–73% of total heat dissipation and was highest for the gyroid architecture. The results demonstrate that increased surface area alone does not ensure improved passive cooling; thermal performance depends on the coupled effects of topology, airflow accessibility and radiative heat exchange. Gyroid TPMS architectures therefore provide a promising DMLS-compatible approach for passive thermal management.

## 1. Introduction

The continuous increase in power density, miniaturisation, and functional integration of modern electronic systems has significantly intensified thermal-management challenges across consumer electronics, power electronics, aerospace, automotive, and renewable-energy applications. As electronic devices become increasingly compact, the dissipation of generated heat becomes a critical design consideration because elevated operating temperatures accelerate material degradation, promote thermo-mechanical fatigue, reduce electrical performance, and ultimately compromise system reliability and service life [1,2,3,4]. Therefore, the development of efficient thermal-management strategies has become essential for ensuring the long-term performance, durability, and operational safety of high-power electronic systems.

Passive cooling technologies remain particularly attractive because they provide reliable heat dissipation without requiring external power, moving components, or active maintenance. Compared with forced-air or liquid-cooling systems, passive heat sinks offer enhanced reliability, lower manufacturing and operational costs, silent operation, and improved energy efficiency, making them well suited to applications where simplicity, robustness, and long-term reliability are significant [2,3,4]. Their thermal performance is evaluated using conjugate heat-transfer computational fluid dynamics (CFD), incorporating conductive, convective and radiative heat-transfer mechanisms.

Heat sinks dissipate thermal energy through three simultaneous heat-transfer mechanisms: conduction within the solid structure, convection between the external surfaces and the surrounding air, and thermal radiation to the ambient environment. Conventional passive heat sinks generally enhance cooling by increasing the available surface area through extended fins, pin-fin arrays, or other protruding geometries while maintaining sufficient spacing to facilitate buoyancy-driven airflow. However, these competing design objectives often lead to conflicting thermal behaviour. Although increasing surface area provides additional heat-transfer area and can improve overall heat dissipation, excessive geometric complexity may restrict airflow, thicken thermal boundary layers, generate stagnant recirculation regions and ultimately reduce natural-convection effectiveness. Therefore, the thermal performance of passive heat sinks operating under natural-convection conditions is governed not simply by surface area but by the complex interaction between heat-transfer area, airflow accessibility, and thermal boundary-layer development [1,2,3,4,5,6].

Similar surface-area and temperature-driving-force considerations arise in conventional heat-exchanger analysis, where heat-transfer performance depends on the available transfer area, thermal resistance and temperature difference between interacting media [7,8].

Although passive heat sinks differ fundamentally from conventional heat exchangers, both systems are governed by conductive and convective heat-transfer principles [7,8]. Increasing effective surface area through fins, lattice architectures or alternative geometric configurations can enhance heat dissipation; however, excessive geometric complexity or insufficient spacing may restrict buoyancy-driven airflow and reduce natural-convection effectiveness. Passive heat-sink optimisation therefore requires an appropriate balance between heat-transfer area and airflow accessibility rather than maximisation of surface area alone [4,7,8,9,10].

Recent advances in additive manufacturing (AM) have fundamentally expanded the design space available for thermal-management components by enabling geometries that are impossible or economically impractical to manufacture using conventional extrusion, casting, or subtractive machining techniques. In particular, laser powder bed fusion technologies such as Direct Metal Laser Sintering (DMLS) enable the fabrication of highly complex lattice structures and triply periodic minimal surface (TPMS) geometries possessing exceptionally high surface-area-to-volume ratios and continuous three-dimensional heat-conduction pathways. Such architectures offer considerable potential for passive cooling because they distribute material efficiently throughout the available volume, promote thermal spreading, and substantially increase the available heat-transfer area [9,11,12].

However, increased geometric complexity does not necessarily translate into improved thermal performance. Dense lattice structures may impede buoyancy-driven airflow, increase flow resistance, promote flow recirculation, and reduce convective heat transfer despite possessing significantly larger nominal surface areas. Recent studies demonstrate that architected metamaterials can tailor functional behaviour through geometric topology, highlighting the broader potential of additively manufactured cellular architectures for engineering applications, including advanced thermal-management systems [4,9,13,14]. Consequently, the thermal behaviour of additively manufactured passive heat sinks is governed by a complex interaction between geometric topology, conductive heat spreading, natural-convection airflow, and radiative heat exchange rather than by surface area alone.

An additional consideration that has received comparatively limited attention is the contribution of thermal radiation. Under natural-convection conditions, radiative heat transfer may account for a substantial proportion of the total heat dissipated by metallic heat sinks, particularly when operating at elevated temperatures and possessing high-emissivity surfaces [2,15,16]. This contribution becomes increasingly important for additively manufactured structures because complex surface morphologies, larger exposed surface areas, and characteristic DMLS surface roughness can significantly influence effective radiative heat transfer. Furthermore, post-processing treatments such as anodising substantially increase the emissivity of aluminium alloys, thereby enhancing radiative cooling performance. Consequently, evaluating passive heat sinks solely based on convective performance may lead to misleading conclusions regarding overall thermal effectiveness. Accurate assessment therefore requires a coupled analysis of conduction, convection, and radiation within a unified thermal framework [6,15,16].

Among the metallic materials currently employed for additively manufactured thermal-management components, AlSi10Mg has emerged as one of the most attractive candidates owing to its favourable combination of low density, relatively high thermal conductivity, excellent corrosion resistance, mechanical integrity, and proven compatibility with DMLS processing. Nevertheless, practical DMLS manufacture introduces important geometric constraints, including minimum wall thickness, unsupported overhang limitations, dimensional tolerances, feature resolution, enclosed-channel accessibility, and surface roughness, all of which influence the manufacturability and thermal performance of complex lattice architectures. As a result, thermally efficient heat-sink designs must also satisfy realistic additive-manufacturing constraints to ensure practical industrial implementation [11,17,18].

Although numerous studies have investigated additively manufactured heat sinks, lattice structures, and TPMS-inspired thermal-management systems, comprehensive comparisons of DMLS-compatible AlSi10Mg passive heat sinks under identical natural-convection conditions remain limited, particularly where conventional finned geometries and lattice/TPMS architectures are evaluated within a consistent conjugate heat-transfer framework that explicitly incorporates thermal radiation. Additionally, the relative contributions of convection and radiation to the overall thermal performance of these architectures remain insufficiently understood. Therefore, it remains unclear whether the enhanced geometric freedom afforded by additive manufacturing genuinely reduces overall thermal resistance or whether increased surface area is offset by diminished buoyancy-driven airflow [4,9,10,13].

To address these knowledge gaps, the present study systematically investigates the thermal performance of five DMLS-compatible AlSi10Mg passive heat-sink architectures subjected to identical natural-convection boundary conditions and a constant thermal load of 18 W. The investigated designs comprise two conventional finned reference geometries—a perforated planar-fin heat sink and a curved radial-fin heat sink—and three advanced additively manufactured architectures consisting of a Schwarz P lattice, a body-centred cubic (BCC) diamond lattice, and a gyroid TPMS structure. Their thermal performance is evaluated using conjugate heat-transfer computational fluid dynamics (CFD), incorporating conductive, convective and radiative heat-transfer mechanisms. Performance is assessed using average and maximum surface temperature, thermal resistance, temperature uniformity, heat-flux distribution, and the relative contributions of convection and radiation to total heat dissipation. The principal novelty of this work lies in the systematic coupled convective–radiative evaluation of DMLS-compatible passive heat-sink architectures within a unified numerical framework. The findings provide new insights into the coupled heat-transfer mechanisms governing passive additively manufactured heat sinks and establish practical design guidelines for optimising next-generation thermal-management systems for high-power electronics and renewable-energy applications [19,20,21].

## 2. Materials and Methods

### 2.1. Study Overview and Thermal Design Framework

This study presents a comparative thermal assessment of five Direct Metal Laser Sintering (DMLS)-compatible AlSi10Mg passive heat-sink architectures under identical thermal loading and ambient conditions. The research focuses on architecture-level thermal performance rather than modelling the DMLS fabrication process. The methodology integrates computer-aided design (CAD), targeted parametric design-space refinement, and computational fluid dynamics (CFD)-based conjugate heat-transfer simulations incorporating conduction, natural convection, and thermal radiation. All architectures were evaluated using consistent material properties, thermal loading, and boundary conditions to enable direct comparison. Performance was quantified using overall thermal resistance, average and maximum surface temperatures, temperature uniformity, heat-flux distribution, and the relative contributions of convective and radiative heat transfer [2,21,22].

### 2.2. Heat Sink Geometries and DMLS Design Constraints

**Figure 1:**
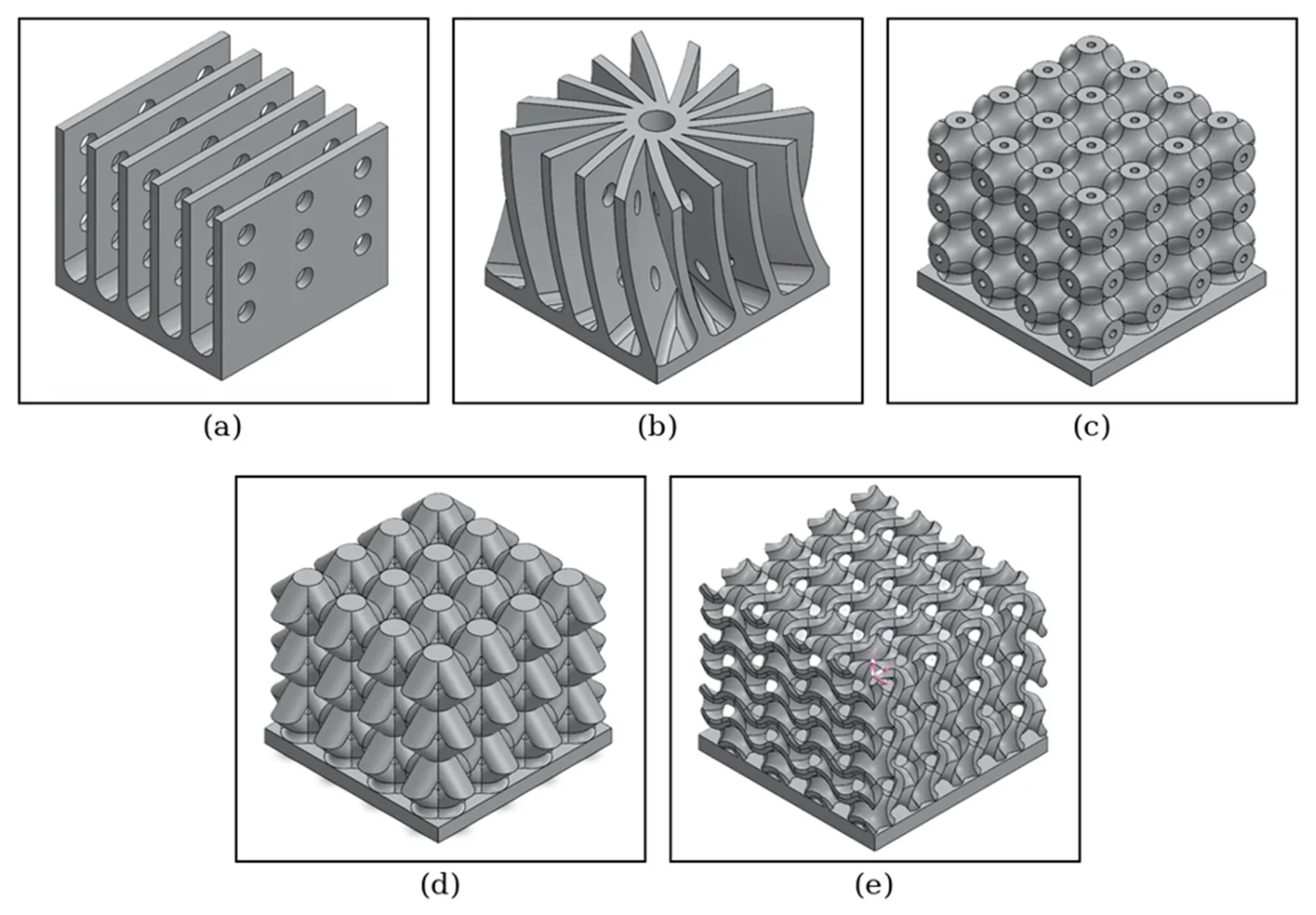
CAD representations of the five passive heat-sink architectures investigated: (**a**) perforated planar-fin; (**b**) curved radial-fin; (**c**) Schwarz P lattice; (**d**) BCC diamond lattice; and (**e**) gyroid lattice.

Five passive heat-sink architectures were studied to evaluate the influence of geometric topology on thermal performance under natural-convection cooling. The selected designs included two conventional fin-based heat sinks and three additive manufacturing (AM)-enabled lattice architectures. The conventional configurations consisted of (1) a perforated planar-fin heat sink and (2) a curved radial-fin heat sink, both representing conventional extended-surface cooling approaches widely used in passive thermal management. The AM-enabled designs included (3) a Schwarz Primitive (Schwarz P) TPMS architecture, (4) a body-centred cubic (BCC) diamond lattice, and (5) a gyroid lattice, each selected to represent architected cellular structures that are either impractical or too expensive to manufacture using conventional machining, extrusion, or casting techniques. The CAD representations of the five investigated heat-sink architectures are presented in Figure 1, illustrating the geometric differences between the conventional fin-based and AM-enabled lattice/TPMS configurations.

This selection enabled direct comparison between conventional fin-based heat sinks, which increase external heat-transfer area, and volumetric lattice architectures that provide high surface-area density and three-dimensional heat-spreading pathways. All geometries were developed according to Design for Additive Manufacturing (DfAM) principles for AlSi10Mg DMLS. Key LPBF constraints, including minimum feature and wall thicknesses, unsupported overhangs, bridge lengths, enclosed cavities, dimensional tolerances, and powder-removal accessibility, were incorporated into the design process. The geometries were therefore configured to minimise unsupported features and support requirements while maintaining geometric integrity and manufacturability, ensuring that the evaluated designs remain practically compatible with DMLS fabrication [17,18,23].

### 2.3. Parametric Geometry Design-Space Refinement

Before the comparative thermal assessment, each heat-sink architecture underwent a targeted parametric design-space refinement to avoid arbitrary or clearly suboptimal configurations. The refinement sought to minimise thermal resistance while maintaining practical DMLS manufacturability. Architecture-specific variables were evaluated within prescribed bounds. For the planar-fin architecture, fin spacing was swept from 2.0 to 10.0 mm in 0.1 mm increments. For the Schwarz P architecture, wall thickness was assessed over 0.8–2.4 mm, while the BCC diamond strut diameter was evaluated over 0.5–6.0 mm. These parameters were selected because they directly influence available heat-transfer area, conductive pathways and airflow accessibility within the respective architectures. The stated ranges are supported by the original design procedure.

The parametric evaluation produced an analytical optimum fin spacing of 5.5 mm for the planar-fin architecture, an analytical wall thickness of 0.8 mm for the Schwarz P architecture, and an upper-bound strut diameter of 6.0 mm for the BCC diamond architecture. These analytical values were subsequently assessed against geometric, thermal and manufacturing considerations rather than adopted automatically as final dimensions.

Accordingly, the planar-fin spacing was adjusted to 5.60 mm, with a fin thickness of 2.00 mm, to accommodate the fixed base dimensions. For the Schwarz P architecture, a final wall thickness of 1.75 mm was selected to provide a practical balance between conductive and radiative heat transfer, internal airflow and manufacturability. For the BCC diamond architecture, a final strut diameter of 5.00 mm was selected because the reduction in thermal resistance diminished above approximately 4 mm, providing a near-optimal compromise between thermal performance, geometric integrity and mass.

The allowable design space was further constrained by DMLS requirements, including minimum feature dimensions, unsupported geometry, structural integrity and powder-removal accessibility. The remaining architecture-specific dimensions, including unit-cell parameters, were prescribed in accordance with these geometric and manufacturing constraints.

MATLAB R2025b was used to perform the bounded parametric evaluations before final CAD reconstruction and CFD assessment. The procedure was therefore treated as a constrained parametric design-space refinement rather than a global mathematical optimisation, providing a consistent basis for comparing DMLS-compatible architectures under identical thermal boundary conditions [4,9,24,25].

### 2.4. Material Selection and Surface Radiative Properties

All heat-sink models were assigned consistent, temperature-independent thermophysical properties of AlSi10Mg, including thermal conductivity, density and specific heat capacity, obtained from published literature [23]. AlSi10Mg was selected because of its established LPBF/DMLS processability, relatively high thermal conductivity, low density and suitability for additively manufactured thermal-management components [24].

Thermal radiation was explicitly coupled with natural convection in all CFD simulations. Heat-sink surfaces were assigned an emissivity value ε = 0.89, which was representative of anodised aluminium to reflect practical surface conditions and enable consistent assessment of convective and radiative heat dissipation. Anodising significantly increases aluminium surface emissivity and can therefore enhance radiative heat rejection under passive cooling conditions [5,15,16].

### 2.5. Computational Domain and Boundary Conditions

**Figure 2:**
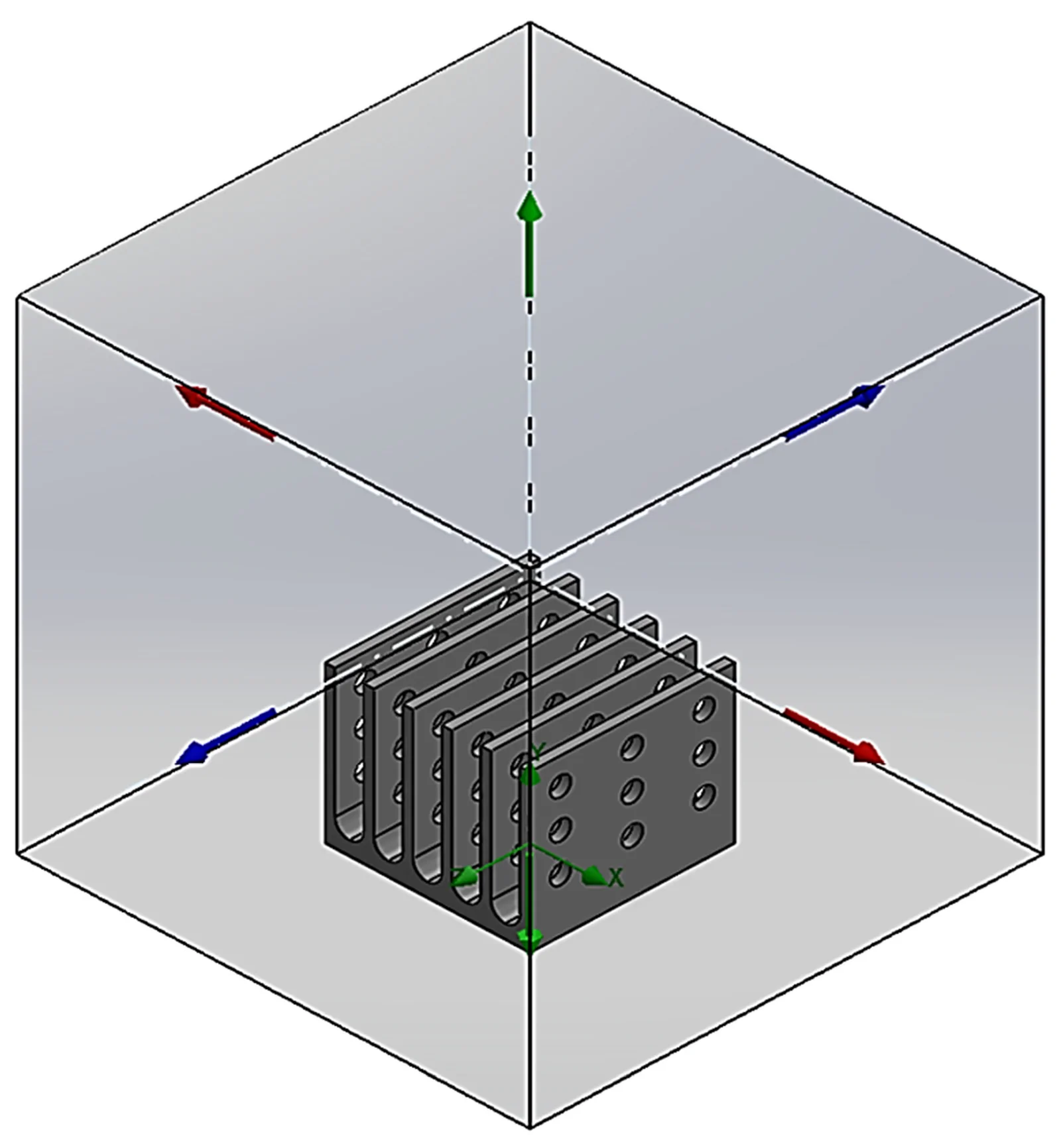
Computational domain to capture buoyancy-driven natural convection and thermal-plume development while limiting boundary influence.

The thermal performance of the five heat-sink architectures was evaluated using SolidWorks Flow Simulation (SWFS) version 2024 under steady-state natural-convection conditions. Each heat sink was enclosed within an external computational domain sufficiently large to permit buoyancy-driven airflow and thermal-plume development while minimising boundary effects (Figure 2). The domain was based on a simplified Silsoe cube methodology, with preliminary sensitivity analysis confirming negligible boundary influence on the predicted thermal performance [20,26].

The surrounding fluid was modelled as quiescent air at ambient room temperature of 25 °C, with a silicon heat source supplying a constant thermal load of 18 W. A conjugate heat-transfer formulation simultaneously resolved conduction through the silicon source and AlSi10Mg heat sink, buoyancy-driven natural convection in the surrounding air, and surface radiation to the ambient environment. Identical material properties, thermal loading and boundary conditions were applied to all architectures to provide a consistent basis for comparison [9,23].

The natural-convection regime was characterised using the Rayleigh (Ra) and Nusselt (Nu) numbers to assess buoyancy-driven flow and convective heat transfer. The calculated dimensionless parameters were used to verify the consistency of the simulated transport behaviour with the applicable laminar natural-convection regime [7,10,27,28].

### 2.6. Governing Physics and Numerical Model

The computational fluid dynamics (CFD) model solved the coupled conservation equations governing mass, momentum and energy transport within the fluid domain together with heat conduction through the solid regions, thereby providing a conjugate heat-transfer solution for the complete heat-sink system. Buoyancy-driven airflow was represented using the Boussinesq approximation, in which density was treated as constant except within the gravitational body-force term of the momentum equation, where temperature-dependent density variations generate natural-convection flow.

The governing equations were discretised using the finite-volume formulation implemented in SolidWorks Flow Simulation. Conduction, natural convection and thermal radiation were solved simultaneously within an external-flow domain. Gravity was specified as 9.81 ms^−2^ in the vertical direction to reproduce buoyancy-driven motion. Both laminar and turbulent flow treatments were enabled within the solver to accommodate local variations in the flow regime. Radiative heat transfer was incorporated through surface-to-surface exchange between the heat-sink surfaces and the surrounding environment. The simulations were performed at an ambient temperature of 25 °C and an environmental pressure of 101,325 Pa, with a surface emissivity of 0.89 and an average surface roughness of 17 μm assigned to the heat-sink surfaces.

The Boussinesq approximation is widely used for passive thermal-management simulations in which temperature-induced density variations remain sufficiently small, providing an efficient representation of buoyancy-driven flow [5,26]. Under the investigated conditions, the resulting thermal behaviour was therefore governed by the coupled interaction of solid conduction, thermal-boundary-layer development, buoyancy-induced plume formation, surface radiation and topology-dependent airflow distribution and recirculation [28,29].

### 2.7. Mesh Independence and Numerical Verification

Numerical uncertainty and model reliability were assessed through mesh-sensitivity analysis (Table 1), analytical radiation verification, global energy-balance assessment and comparison with established natural-convection correlations. Mesh refinement from levels 3–6 produced successive variations in maximum temperature of 0.40%, 0.74% and 0.55%. Considering the limited variation with further refinement and the associated computational cost, a global refinement level of 4.0, with a local refinement ratio factor of 0, was applied consistently to all five architectures [27,30].

Radiative heat-transfer predictions were independently verified using the Stefan–Boltzmann relation, with deviations of 1.48–4.34% across the five architectures. The global energy imbalance remained below approximately 1 W for the prescribed 18 W heat input, while Rayleigh-number calculations confirmed consistency with the applicable natural-convection flow regime. The adopted surface emissivity of 0.89 lies within the reported range of 0.88–0.90 for anodised aluminium, while the AlSi10Mg thermal conductivity of 160 W m^−1^ K^−1^ represents the midpoint of the reported directional range of 155–165 W m^−1^ K^−1^. Collectively, these assessments support the numerical reliability of the predicted comparative thermal trends [7,10,31].

### 2.8. Performance Metrics

The primary performance metric was the overall thermal resistance (Rth), defined as the temperature rise above ambient per unit heat input. Lower Rth indicates greater overall heat-dissipation effectiveness under identical operating conditions [2,7,8].

Additional metrics included average, maximum and minimum surface temperatures, temperature uniformity, heat-flux distribution, convective and radiative heat-transfer contributions, heat-transfer coefficients, heat-sink mass and estimated manufacturing cost. These parameters were evaluated consistently across all architectures to quantify thermal performance, temperature distribution and heat-transfer mechanisms while enabling comparison of the practical implications of conventional fin-based and lattice/TPMS designs [4,24,32].

## 3. Results

### 3.1. Surface Temperature and Temperature Uniformity

**Figure 3:**
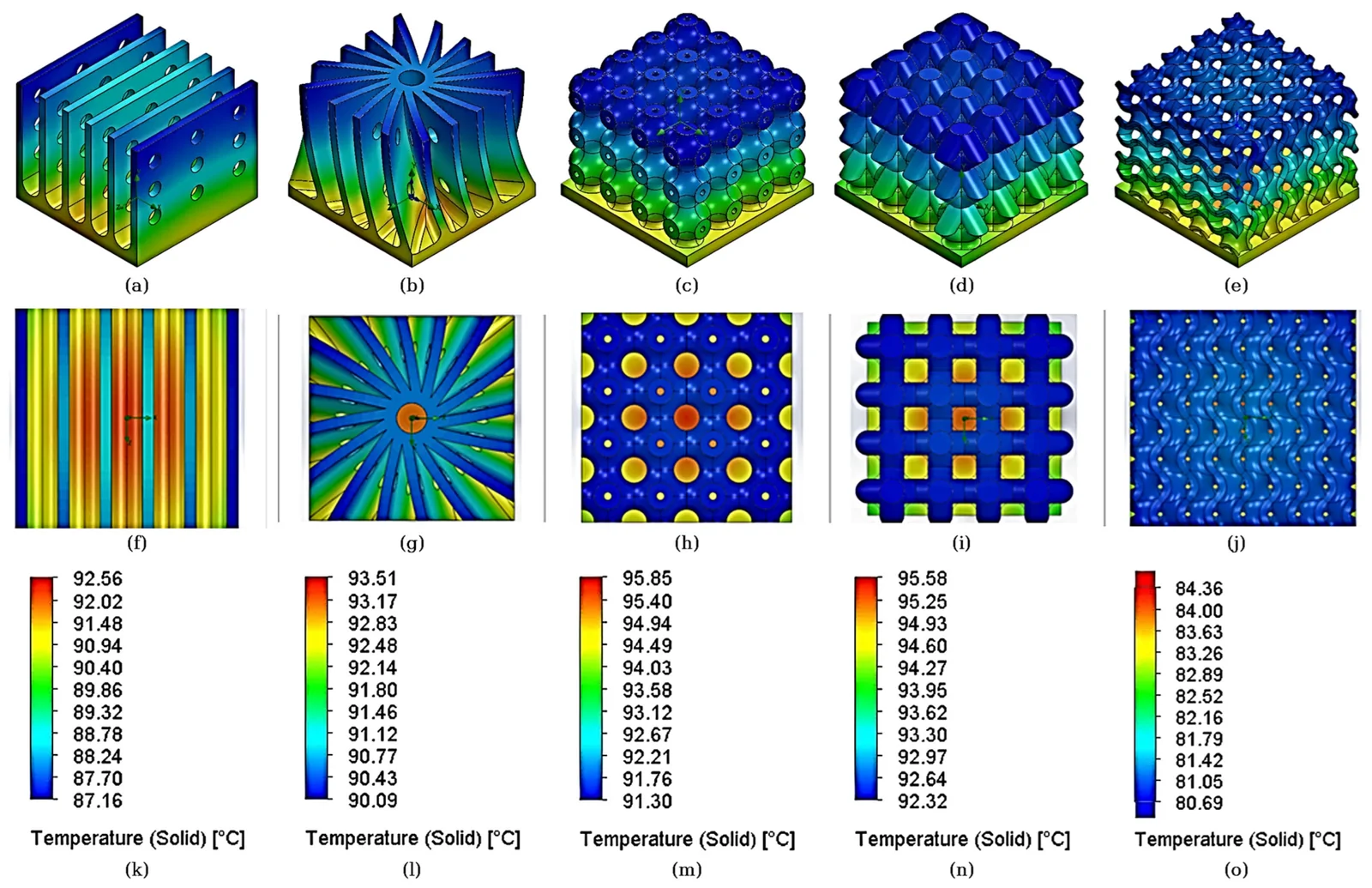
Surface temperature distributions: (**a**–**e**) 3D views and (**f**–**j**) plan views of (**a**,**f**) perforated planar-fin; (**b**,**g**) curved radial-fin; (**c**,**h**) Schwarz P; (**d**,**i**) BCC diamond; and (**e**,**j**) gyroid heat sinks; (**k**–**o**) corresponding temperature scales.

Figure 3 illustrates the surface temperature distributions for the five heat-sink architectures, providing a direct visual comparison of their thermal behaviour under identical operating conditions. The three-dimensional temperature fields (Figure 3a–e) together with the corresponding plan-view temperature maps (Figure 3f–j) show substantial differences in both heat-removal capability and temperature uniformity. Among the studied designs, the gyroid lattice (Figure 3e,j) shows the lowest surface temperatures, with values ranging from approximately 78.2 to 84.2 °C, indicating the most effective overall heat dissipation. The predominantly blue temperature field, interrupted only by small isolated warmer regions, suggests efficient heat spreading throughout the interconnected TPMS structure and enhanced interaction with buoyancy-driven airflow [13,19,24].

In contrast, the perforated planar-fin heat sink (Figure 3a,f) displays a pronounced longitudinal temperature gradient, with a distinct hot central region and progressively cooler outer fins. This behaviour indicates that heat is preferentially conducted along individual fins, resulting in less effective lateral heat spreading despite the relatively low maximum temperature. Similarly, the curved radial-fin configuration (Figure 3b,g) demonstrates a central hot spot located directly above the heat source, from which temperature decreases radially towards the fin tips. Although the radial geometry improves multidirectional conduction, the persistent central thermal concentration indicates that the available convective cooling is insufficient to eliminate localised heat accumulation [3,29].

The Schwarz P (Figure 3c,h) and BCC diamond (Figure 3d,i) lattice structures show comparatively higher average temperatures, with peak values approaching 95.6 °C. However, both architectures show highly homogeneous temperature distributions over their upper surfaces, characterised by relatively smooth thermal gradients and minimal localised hot spots. This behaviour indicates relatively uniform thermal spreading within the lattice networks but comparatively lower overall heat-removal capability than the gyroid configuration. The increased average temperature suggests that, despite their uniform heat distribution, these periodic lattice structures offer less favourable airflow pathways and reduced convective heat transfer under natural convection conditions.

Overall, the study demonstrates that thermal performance cannot be assessed using average surface temperature alone. While the gyroid architecture provides the greatest heat-removal capability by maintaining the lowest operating temperatures, the Schwarz P and BCC diamond structures achieve superior thermal uniformity through more even heat distribution. These observations confirm that temperature uniformity and absolute heat-removal capacity represent complementary performance metrics rather than equivalent measures of thermal effectiveness. Consequently, both should be evaluated concurrently when assessing passive heat-sink architectures, particularly for applications where minimising thermal gradients and avoiding localised hot spots are as important as reducing the overall operating temperature [13,19,24,29].

The temperature uniformity index (UI) quantifies the degree of spatial temperature variation across the heat-sink surface. Lower UI values indicate a more uniform temperature distribution and smaller thermal gradients, whereas higher values correspond to greater temperature non-uniformity and an increased likelihood of localised hot spots and thermally induced stresses.

**Figure 4:**
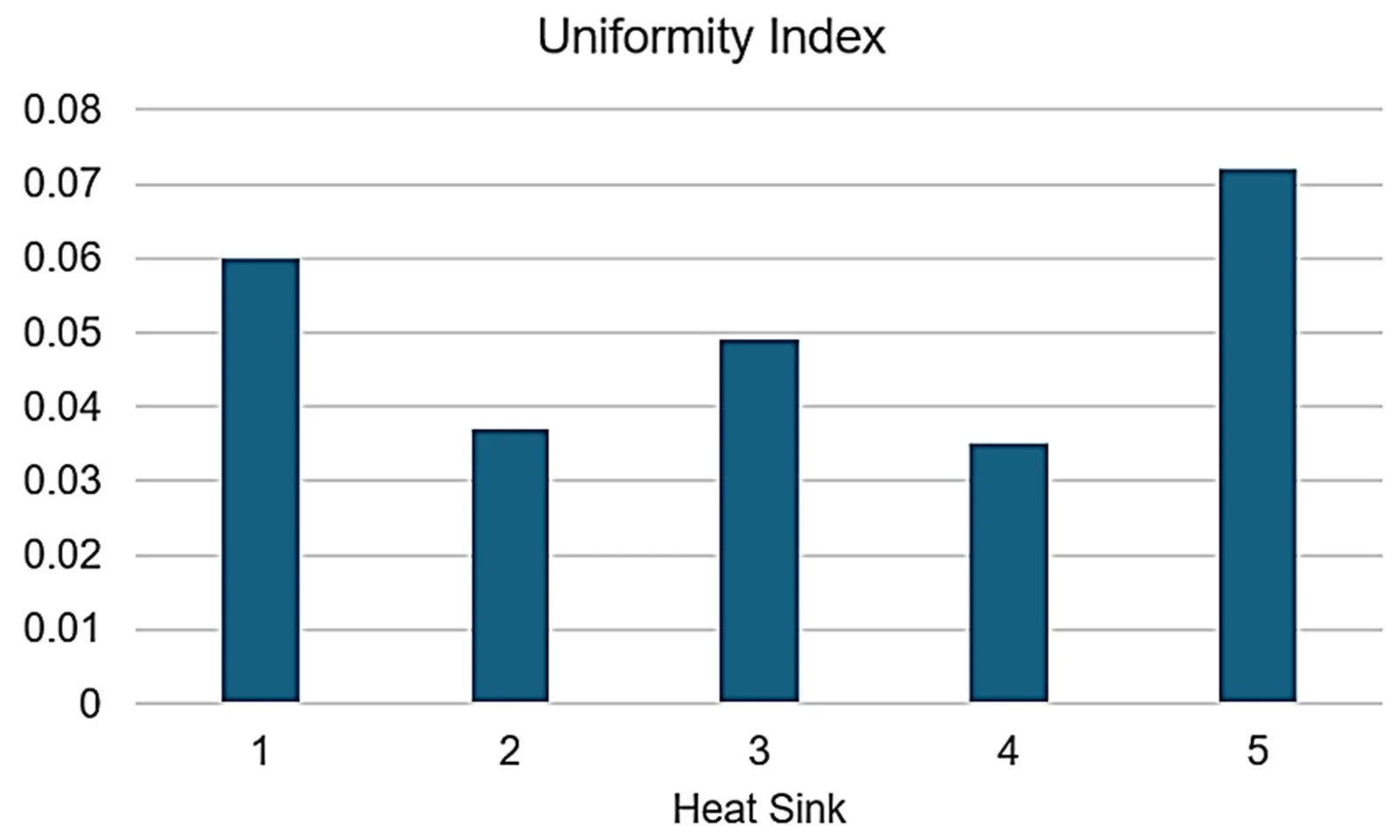
Temperature uniformity comparison. The BCC diamond lattice achieved the lowest uniformity index, while the gyroid produced the lowest average temperature but not the most uniform distribution.

The qualitative observations from the temperature contours are corroborated by the quantitative data presented in Table 2 and Figure 4. The gyroid heat sink (Heat Sink 5) achieved the lowest average surface temperature (82.02 °C), together with the lowest maximum (84.17 °C) and minimum (78.23 °C) temperatures, demonstrating the highest overall heat-dissipation capability among the five architectures. The substantial reduction in operating temperature compared with the conventional finned and lattice designs indicates that the interconnected TPMS geometry promotes more effective heat transfer under natural-convection conditions.

However, the gyroid did not exhibit the most uniform surface temperature distribution. Instead, it produced the largest temperature uniformity index (UI = 0.072), reflecting a wider temperature spread across the heat-sink surface despite maintaining the lowest overall temperatures. This behaviour is consistent with the temperature contours shown in Figure 3e,j, where efficient heat removal is accompanied by localised temperature variations associated with the complex three-dimensional flow passages.

In contrast, the BCC diamond lattice (Heat Sink 4) achieved the lowest temperature uniformity index (UI = 0.035), followed closely by the curved radial-fin heat sink (Heat Sink 2, UI = 0.037). These lower values indicate a more homogeneous surface temperature distribution and reduced thermal gradients, despite their higher average temperatures of 93.30 °C and 91.39 °C, respectively. Similarly, the Schwarz P lattice (Heat Sink 3) exhibited a relatively uniform thermal field (UI = 0.049) but operated at an even higher average temperature (92.83 °C). These findings demonstrate that enhanced thermal spreading within periodic lattice structures does not necessarily translate into superior heat-removal capability.

The perforated planar-fin heat sink (Heat Sink 1) exhibited an intermediate average temperature of 89.36 °C but a comparatively higher uniformity index (UI = 0.060), indicating the presence of stronger temperature gradients along the fin length. This observation agrees with the contour plots, which reveal preferential heat conduction along individual fins and the development of a central hot region due to limited lateral heat spreading.

The combined analysis demonstrates that average operating temperature and temperature uniformity characterise different aspects of thermal performance. While the gyroid architecture is the most effective at removing heat and maintaining the lowest operating temperatures, the BCC diamond lattice provides the most spatially uniform temperature distribution. Consequently, the optimum heat-sink architecture depends on the design objective: applications prioritising maximum heat dissipation benefit from the gyroid topology, whereas systems sensitive to thermal gradients and thermally induced stresses may favour more temperature-uniform architectures such as the BCC diamond lattice. This highlights the importance of employing multiple performance metrics rather than relying solely on average temperature or thermal resistance when evaluating advanced passive heat-sink designs [13,19,33,34].

### 3.2. Flow Behaviour Under Natural Convection

**Figure 5:**
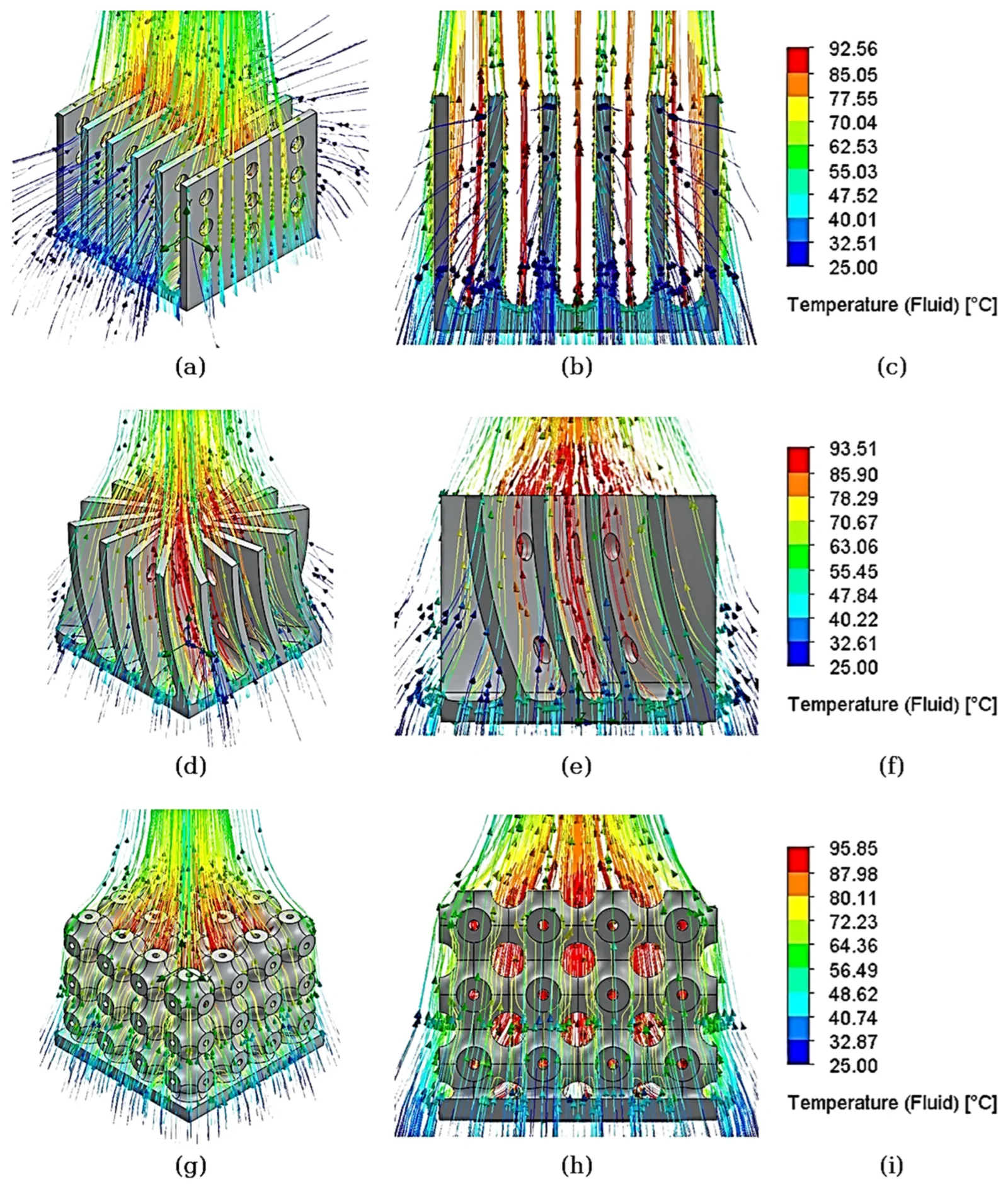
Simulated natural-convection flow behaviour for Heat Sinks 1–3. The visualisations illustrate the contrast between organised channel flow in the finned design and more complex plume behaviour in the lattice geometry. (**a**–**c**) perforated planar-fin heat sink; (**d**–**f**) curved radial-fin heat sink; (**g**–**i**) Schwarz P lattice.

**Figure 6:**
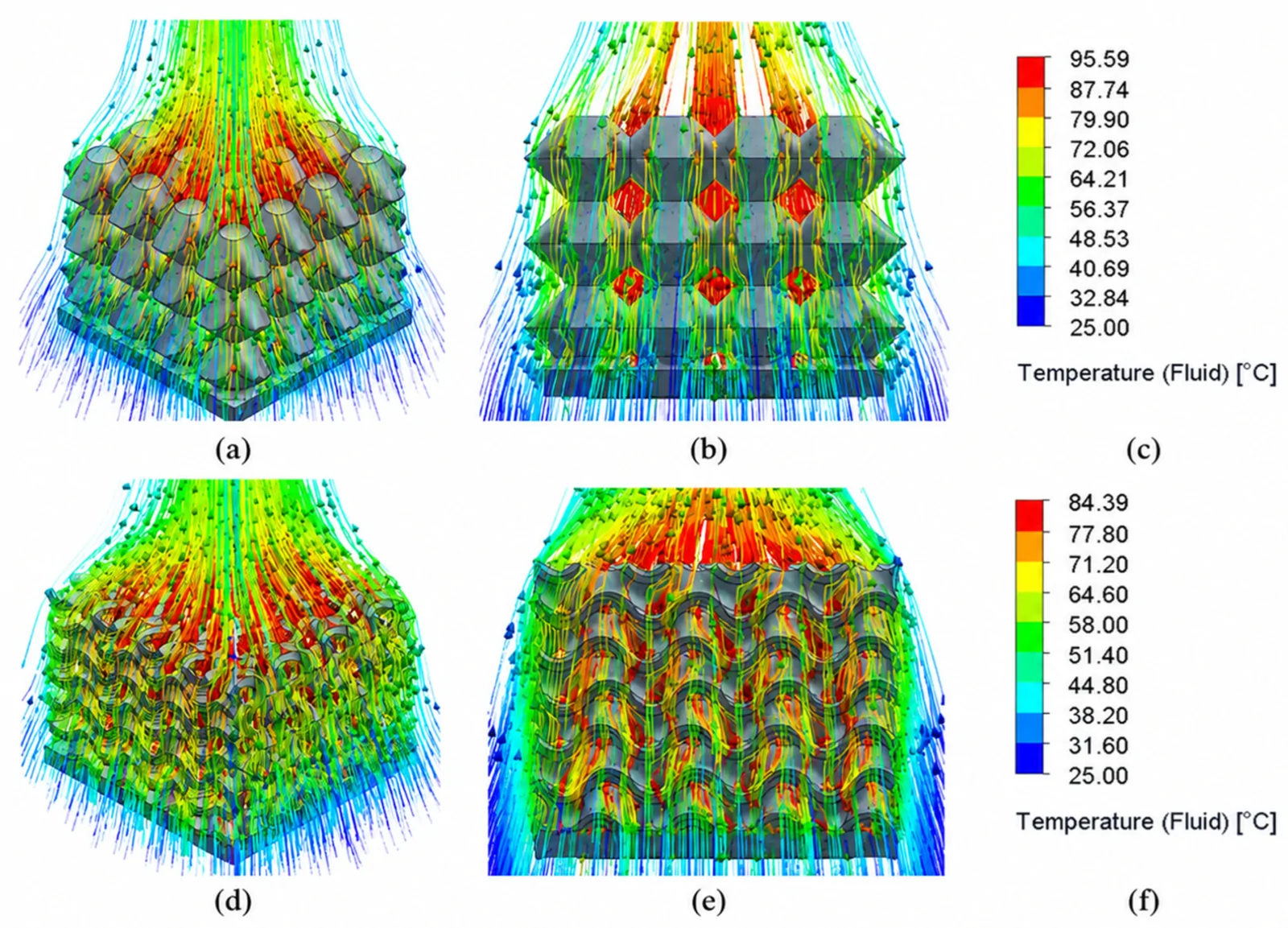
Simulated natural-convection flow behaviour: (**a**) BCC diamond lattice, 3D flow field; (**b**) BCC diamond lattice, sectional flow field; (**c**) corresponding fluid-temperature scale; (**d**) gyroid lattice, 3D flow field; (**e**) gyroid lattice, sectional flow field; (**f**) corresponding fluid-temperature scale. Lines and directional symbols indicate airflow streamlines and local flow direction.

The simulated natural-convection flow fields presented in Figure 5 and Figure 6 provide further insight into the mechanisms responsible for the observed differences in thermal performance among the five heat-sink architectures. While increasing surface area is generally beneficial for passive cooling, the effectiveness of this additional area depends on its accessibility to buoyancy-driven airflow. The streamlines demonstrate that heat-sink geometry strongly influences the formation, continuity and strength of the thermal plume, which ultimately governs the efficiency of convective heat removal [19].

The perforated planar-fin heat sink (Figure 5a,b) exhibits well-defined vertical airflow channels between adjacent fins. Heated air rises almost uniformly through these open passages, while cooler ambient air is entrained from the sides and base of the heat sink. The fin perforations further promote cross-flow between neighbouring channels, reducing stagnant regions and improving the replenishment of cooler air. These organised flow paths explain the relatively effective convective cooling observed for this conventional design [10,34].

The curved radial-fin heat sink (Figure 5d,e) also promotes strong buoyancy-driven flow; however, the radial fin arrangement causes the thermal plume to diverge outward from the central heat source before merging into a single upward plume. This multidirectional flow enhances air mixing around the outer fin surfaces but produces a region of accelerated flow convergence above the centre of the heat sink. The corresponding temperature contours show that this region coincides with the localised hot spot previously identified on the upper surface, indicating that the enhanced convection is insufficient to eliminate the concentration of heat directly above the heat source [13].

The Schwarz P lattice (Figure 5g,h) displays a markedly different flow behaviour. Although the periodic lattice substantially increases the available heat-transfer area, the interconnected cellular structure forces the buoyant airflow through narrow and tortuous internal passages. The flow visualisation reveals numerous regions of reduced velocity and local recirculation within the lattice network, limiting the continuous renewal of cooler ambient air over portions of the internal surfaces. Consequently, part of the additional surface area becomes less effective for convective heat transfer, contributing to the higher average operating temperature observed for this architecture.

A similar trend is evident for the BCC diamond lattice (Figure 6a,b). The dense three-dimensional strut network produces a relatively homogeneous thermal plume above the heat sink; however, the closely spaced lattice elements impede airflow penetration into the interior of the structure. The streamlines indicate that much of the buoyancy-driven flow is diverted around the outer surfaces rather than uniformly distributed throughout the internal volume. Although this architecture exhibits excellent thermal spreading and the lowest temperature uniformity index, the restricted airflow limits its overall convective heat-removal capability.

In contrast, the gyroid lattice (Figure 6d,e) exhibits the most favourable flow characteristics. The continuously interconnected triply periodic minimal surface (TPMS) provides smooth, curvature-driven pathways that allow the buoyancy-induced airflow to penetrate more uniformly throughout the structure. Unlike the discrete cellular lattices, the absence of sharp junctions and enclosed cavities reduces flow separation and promotes continuous upward movement of heated air. The streamlines demonstrate a more evenly distributed thermal plume across the entire geometry, enabling more effective utilisation of the extensive internal surface area. This behaviour is consistent with the significantly lower fluid temperatures surrounding the heat sink and explains why the gyroid architecture achieved the lowest average surface temperature and thermal resistance among all the configurations investigated.

The flow visualisations demonstrate that passive cooling performance depends on achieving an appropriate balance between surface area and airflow accessibility. While the Schwarz P and BCC diamond lattices provide substantial increases in surface area, their complex internal networks introduce additional hydraulic resistance and restrict natural-convection flow. In contrast, the perforated planar-fin and curved radial-fin geometries maintain relatively unobstructed flow channels, whereas the gyroid lattice successfully combines a large heat-transfer surface with continuous interconnected flow pathways. These observations confirm that maximising surface area alone is insufficient for optimal passive cooling; rather, the internal geometry must also facilitate efficient buoyancy-driven airflow to fully exploit the available heat-transfer area [13,19,22,35].

### 3.3. Heat Flux Distribution

**Figure 7:**
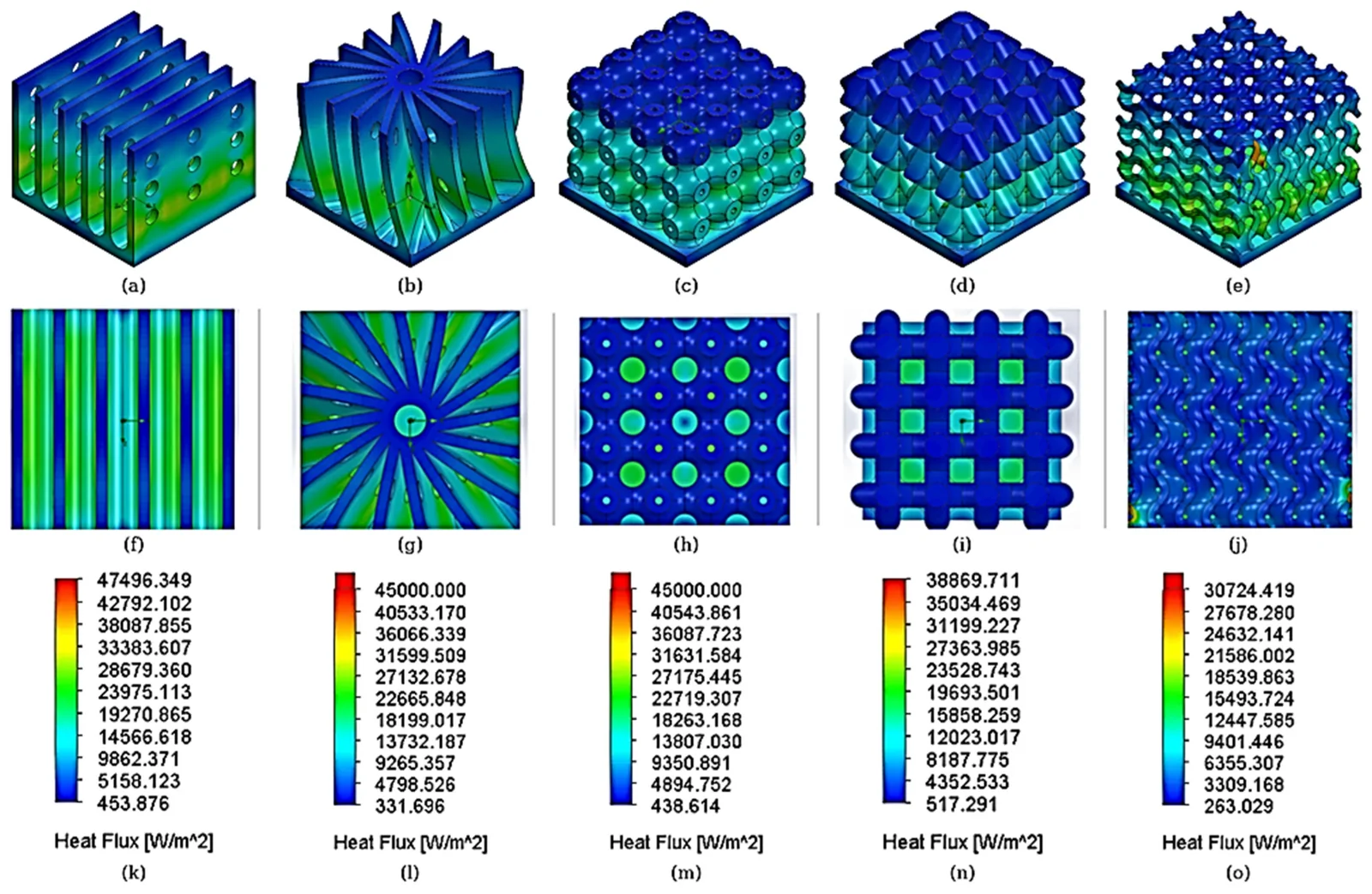
Heat-flux distributions: (**a**–**e**) 3D views and (**f**–**j**) plan views of (**a**,**f**) perforated planar-fin; (**b**,**g**) curved radial-fin; (**c**,**h**) Schwarz P; (**d**,**i**) BCC diamond; and (**e**,**j**) gyroid heat sinks; (**k**–**o**) corresponding heat-flux scales.

**Figure 8:**
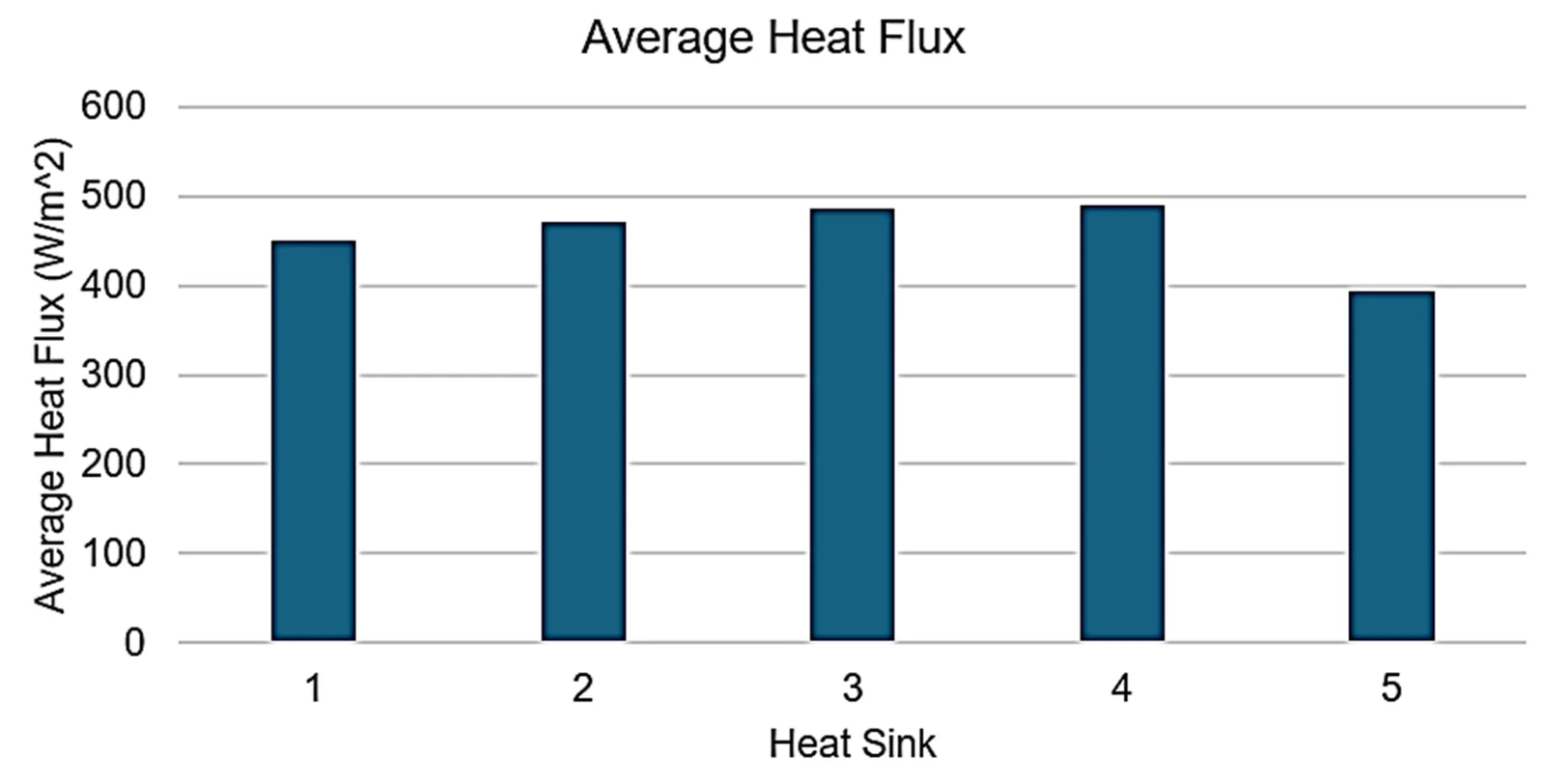
Average heat flux comparison for the five heat-sink geometries.

The heat-flux distributions shown in Figure 7, together with the quantitative results summarised in Table 3 and Figure 8, provide further understanding of the thermal behaviour of the five passive heat-sink architectures. Since all geometries were subjected to the same heat input of 18 W, differences in average heat flux arise from variations in thermal spreading, effective surface utilisation, and the accessibility of the heat-transfer surfaces to buoyancy-driven airflow.

The perforated planar-fin heat sink (Figure 7a,f) shows distinct longitudinal bands of elevated heat flux concentrated along the central fin surfaces, with progressively lower values towards the outer fins.

This behaviour indicates that heat is preferentially conducted through the central fin regions before being dissipated to the surrounding air by natural convection. Similarly, the curved radial-fin heat sink (Figure 7b,g) displays a localised region of high heat flux surrounding the central hub, from which thermal energy is distributed radially along the fins. Although the radial arrangement promotes multidirectional heat spreading, the concentration of heat near the heat source remains evident, consistent with the temperature distributions observed in Figure 3 [36,37].

The Schwarz P lattice (Figure 7c,h) and the BCC diamond lattice (Figure 7d,i) show more heterogeneous heat-flux fields, with elevated heat flux concentrated around the interconnected lattice nodes and primary structural members. These localised regions indicate that thermal energy is preferentially transferred through selected conduction pathways, while portions of the internal surface remain less effective because the dense lattice networks restrict the penetration of buoyancy-driven airflow. This interpretation is supported by the natural-convection flow visualisations presented in Figure 5 and Figure 6, where both lattice architectures show more complex internal flow paths and reduced airflow accessibility compared with the finned configurations [37,38].

In contrast, the gyroid lattice (Figure 7e,j) exhibits a more homogeneous heat-flux distribution with substantially lower local heat-flux concentrations across its continuously interconnected triply periodic minimal surface (TPMS). The smoother heat-flux field indicates that thermal energy is distributed more uniformly throughout the structure before being transferred to the surrounding fluid. This behaviour is consistent with the enhanced airflow penetration observed in Figure 6 and the lower surface temperatures reported in Figure 3, demonstrating more effective utilisation of the available heat-transfer surface.

The quantitative comparison shown in Table 3 and Figure 8 further supports these observations. The BCC diamond lattice exhibited the highest average heat flux (490.42 W m^−2^), followed by the Schwarz P lattice (487.00 W m^−2^), the curved radial-fin heat sink (472.01 W m^−2^), and the perforated planar-fin heat sink (449.94 W m^−2^). In contrast, the gyroid lattice produced the lowest average heat flux (393.39 W m^−2^), representing reductions of approximately 20% and 19% relative to the BCC diamond and Schwarz P lattices, respectively. Although lower heat-flux values are often interpreted as reduced heat-transfer intensity, in this case, they reflect a more effective distribution of the imposed thermal load over a larger actively cooled surface. Therefore, local thermal loading is reduced while the overall heat-removal capability is enhanced [38,39].

When considered alongside the temperature contours (Figure 3), the temperature uniformity analysis (Table 2), and the natural-convection flow fields (Figure 5 and Figure 6), the heat-flux results confirm that thermal performance cannot be attributed solely to surface area. While the Schwarz P and BCC diamond lattices possess extensive internal surface area, their relatively dense cellular networks reduce airflow permeability and limit the effective utilisation of that surface for convective heat transfer. In contrast, the gyroid architecture combines a large, interconnected heat-transfer surface with continuous airflow pathways, enabling more uniform thermal spreading, lower local heat-flux concentrations, and superior passive cooling performance. These findings demonstrate that geometric topology, airflow accessibility, and effective surface utilisation are equally important design parameters for the optimisation of passive heat sinks operating under natural-convection conditions [39,40,41].

### 3.4. Radiative and Convective Contributions

**Figure 9:**
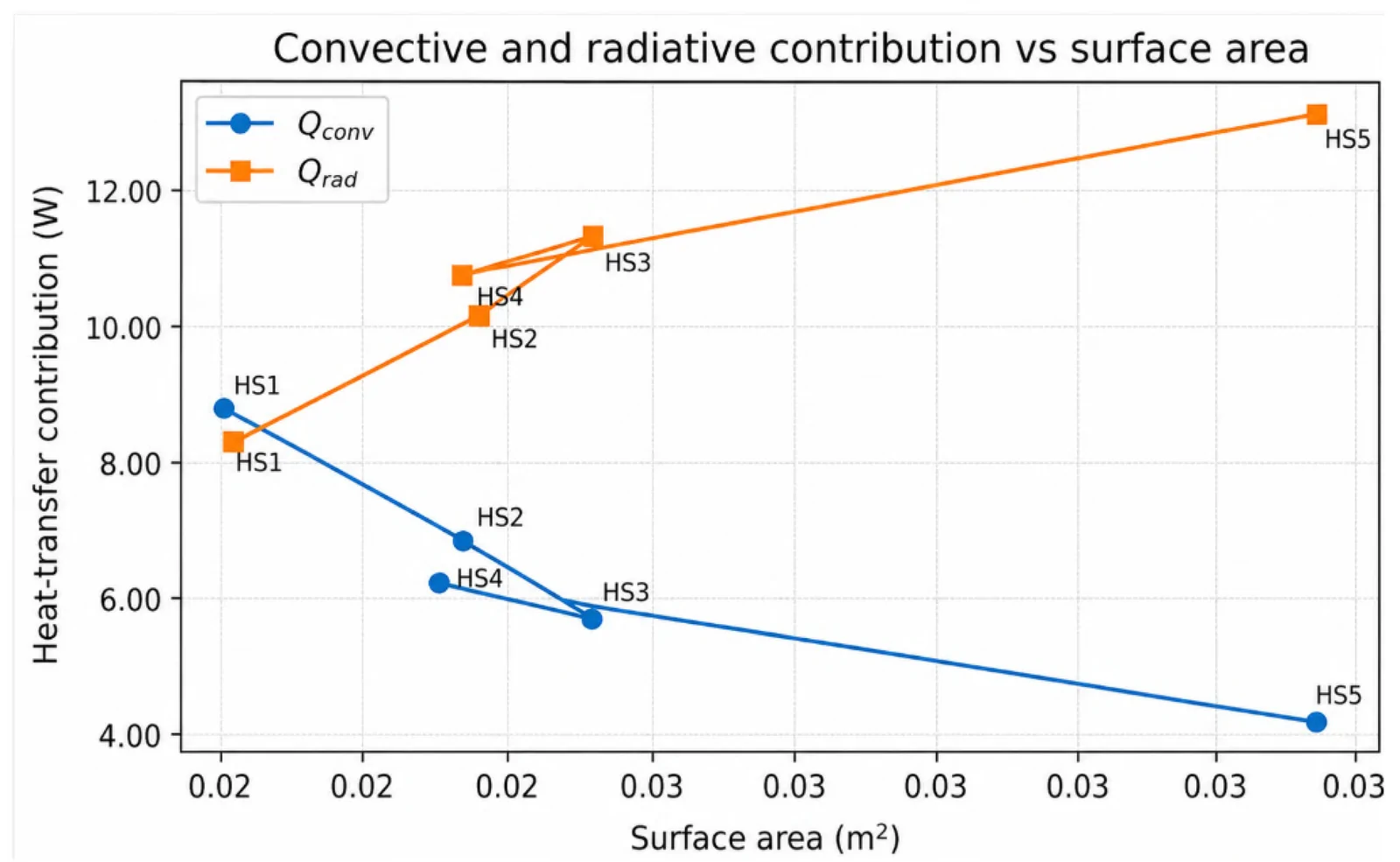
Relationship between surface area and heat-transfer coefficients. Convective performance declined strongly in the gyroid case despite its large surface area, whereas radiative performance remained more closely linked to exposed area and surface temperature.

The relative contributions of convection and thermal radiation to the total heat dissipation are summarised in Table 4, while the influence of surface area on the extracted heat-transfer coefficients is presented in Table 5 and Figure 9. The results demonstrate that thermal radiation constitutes a significant component of the overall heat-transfer mechanism for all five passive heat-sink architectures. Radiation contributed between 46.33% and 72.52% of the total heat rejected, indicating that radiative heat transfer cannot be neglected when evaluating metallic heat sinks operating under natural-convection conditions.

The perforated planar-fin heat sink exhibited the highest convective contribution, with convection accounting for 48.94% of the total heat dissipation, while radiation contributed 46.33%. This behaviour is consistent with the natural-convection flow fields presented in Figure 5, where the open parallel-fin channels promote unobstructed buoyancy-driven airflow and continuous entrainment of cooler ambient air. The efficient airflow through the fin passages enhances convective heat transfer, resulting in the largest convective heat-transfer coefficient among the five configurations (7.43 W m^−2^ K^−1^), as reported in Table 5.

The curved radial-fin heat sink exhibited a more balanced heat-transfer mechanism, with radiation contributing 56.61% and convection 38.00% of the total heat dissipation. Although the radial fin arrangement supports multidirectional airflow, the convergence of fins near the centre restricts local air movement above the heat source, reducing the effectiveness of convection relative to the perforated planar-fin configuration. Consequently, radiative heat transfer assumes a more dominant role despite the relatively modest increase in surface area.

For the Schwarz P and BCC diamond lattice architectures, radiation accounted for 63.01% and 59.84%, respectively, whereas convection contributed only 31.69% and 34.67%. These trends are consistent with the flow visualisations shown in Figure 5 and Figure 6, which revealed increasingly tortuous internal flow paths and reduced airflow penetration within the dense lattice structures. Although these architectures possess larger heat-transfer surface areas than the conventional finned designs, much of the internal surface is less effectively utilised because buoyancy-driven airflow is impeded by the complex cellular networks. Thus, convection becomes progressively less effective, while radiation assumes a greater proportion of the total heat-transfer process [38,41].

The gyroid architecture provides improved airflow accessibility and more effective utilisation of its extensive heat-transfer surface, while its overall superior cooling performance results from the combined effects of thermal spreading, convection and dominant radiative heat transfer. As shown in Table 4, radiation contributed 72.52% of the total heat dissipation, whereas convection accounted for only 23.43%. Despite possessing the largest surface area (0.0330 m^2^) of all the investigated architectures (Table 5), the gyroid exhibited the lowest convective heat-transfer coefficient (2.24 W m^−2^ K^−1^). At first glance, this observation may appear counterintuitive; however, it reflects the reduction in average surface-to-ambient temperature difference associated with the significantly lower operating temperature of the gyroid (82.02 °C). As demonstrated previously by the temperature distributions (Figure 3), the flow visualisations (Figure 6), and the heat-flux analysis (Figure 7 and Figure 8), the continuously interconnected TPMS geometry distributes thermal energy more uniformly throughout the structure while maintaining effective airflow accessibility.

Subsequently, the imposed thermal load is distributed over a larger effective surface area and a smaller mean surface-to-ambient temperature difference, resulting in a lower area-averaged convective heat-transfer coefficient despite the superior overall cooling performance [38,39,40,41].

Figure 9 further illustrates the relationship between surface area and the extracted heat-transfer coefficients. Increasing surface area does not produce a proportional increase in the convective heat-transfer coefficient. Instead, the results reveal a general reduction in the calculated convective coefficient as surface area increases from the perforated planar-fin geometry to the gyroid lattice. This behaviour indicates that increases in internal surface area do not translate proportionally into higher area-averaged convective coefficients, because local airflow accessibility, velocity and thermal-boundary-layer development vary with topology.

On the other hand, the radiative heat-transfer coefficient remains comparatively stable across all five architectures, varying only between approximately 6.93 and 7.37 W m^−2^ K^−1^ (Table 5). Unlike convection, thermal radiation depends primarily on surface temperature, emissivity and the effective radiating area, and is therefore less sensitive to internal airflow restrictions. Consequently, as the architectural complexity and surface area increase, radiation contributes an increasingly significant proportion of the total heat dissipation, partially compensating for the reduction in convective effectiveness [38,41].

When considered together with the temperature contours (Figure 3), temperature uniformity (Table 2), airflow visualisations (Figure 5 and Figure 6), and heat-flux distributions (Figure 7 and Figure 8), these findings demonstrate that passive heat-sink performance is governed by the combined interaction of conduction, natural convection and thermal radiation. Although complex lattice and TPMS geometries substantially increase surface area, their thermal effectiveness depends on maintaining sufficient airflow accessibility while simultaneously maximising radiative heat exchange. Among the architectures investigated, the gyroid lattice provides the most favourable balance of these mechanisms, combining extensive heat-transfer surface area with improved thermal spreading and a dominant radiative contribution to achieve the lowest operating temperature and thermal resistance under passive cooling conditions [38,39,40].

### 3.5. Thermal Resistance and Design Efficiency

The combined effects of natural convection and thermal radiation on thermal performance are summarised in Table 6. Unlike the individual heat-transfer coefficients presented in Table 5, the combined thermal resistance represents the overall effectiveness of each architecture in dissipating the imposed 18 W heat load by accounting for the simultaneous contributions of conduction, convection, and radiation.

The gyroid lattice achieved the lowest combined thermal resistance (3.30 °C W^−1^), demonstrating the highest overall cooling performance. This finding is consistent with the lowest average surface temperature (Figure 3), the more uniform heat-flux distribution (Figure 7 and Figure 8), and the continuous buoyancy-driven airflow observed in Figure 6. Although the gyroid shows the lowest convective heat-transfer coefficient, its large interconnected TPMS surface and the highest radiative contribution (72.52%, Table 4) more than compensated, resulting in the most effective passive heat dissipation. On the other hand, the BCC diamond lattice produced the highest combined thermal resistance (4.01 °C W^−1^) despite its relatively large surface area. As shown in Figure 6, the dense strut network restricts airflow through the structure, leading to localised heat-flux concentrations (Figure 7 and Figure 8) and reduced utilisation of the available heat-transfer surface. These results demonstrate that increasing geometric complexity alone does not necessarily improve passive cooling performance.

The perforated planar-fin and curved radial-fin heat sinks exhibited intermediate thermal resistances of 3.75 °C W^−1^ and 3.89 °C W^−1^, respectively. Their open flow channels (Figure 5) enhance buoyancy-driven convection, but their smaller surface areas reduce the contribution of thermal radiation compared with the gyroid. Similarly, the Schwarz P lattice achieved a thermal resistance of 3.99 °C W^−1^, reflecting the trade-off between increased surface area and restricted internal airflow.

Overall, the results demonstrate that passive thermal performance is governed by the coupled effects of surface area, airflow accessibility, thermal spreading, and convective–radiative heat exchange. The superior performance of the gyroid is attributed to its continuous interconnected topology, which combines high heat-transfer surface area with favourable internal transport pathways, consistent with previous studies demonstrating the potential of TPMS architectures for advanced thermal management [38,41].

The practical implications of the different architectures were further assessed through estimated mass and material cost (Table 7). The BCC diamond exhibited the highest mass and cost, whereas the perforated planar-fin design was the lightest and least expensive, highlighting the trade-off between thermal performance, geometric complexity and material utilisation.

### 3.6. Numerical Verification

The numerical model was verified by comparing the simulated radiative heat-transfer rates with analytical predictions based on the Stefan–Boltzmann equation.

(1) $$Q_{rad}=\varepsilon \sigma A\left(T_{s}^{4}-T_{\infty }^{4}\right)$$

where ε  is the surface emissivity, $\sigma =5.67\times 10^{-8}\;W\;m^{-2}\;K^{-4}$ is the Stefan–Boltzmann constant, A is the radiating surface area, and $T_{s}\;$ and $T_{\infty }\;$ are the absolute surface and ambient temperatures, respectively. The percentage error was calculated as

(2) $$\%Error=\frac{|Q_{sim}-Q_{calc}|}{Q_{sim}}\times 100$$

In Table 8, the comparison of the simulated and analytical radiative heat-transfer rates for the five heat-sink architectures shows good agreement, with percentage differences ranging from 1.48% to 4.34%, demonstrating that the numerical radiation model accurately reproduces the theoretical predictions. The small deviations are expected because the analytical solution assumes uniform surface temperature and simplified radiative exchange, whereas the CFD model resolves the complete three-dimensional temperature distribution and surface interactions.

The numerical solution was further verified through an energy-balance assessment.

(3) $$Q_{in}\approx Q_{conv}+Q_{rad}$$

where $Q_{in}$ is the imposed 18 W heat input, and $Q_{conv}$ and $Q_{rad}$ are the corresponding convective and radiative heat-loss rates, respectively. In addition, the calculated Rayleigh numbers remained within the laminar natural-convection regime, confirming that the selected flow model and boundary conditions were appropriate for all configurations.

The close agreement between analytical and numerical predictions, together with satisfactory energy conservation and flow-regime verification, provides confidence in the accuracy of the CFD methodology for evaluating the thermal performance of the perforated planar-fin, curved radial-fin, Schwarz P lattice, BCC diamond lattice, and gyroid lattice heat sinks.

In addition to the present analytical and numerical verification procedures, the adopted computational methodology is supported by published experimental–numerical investigations of naturally cooled heat sinks [36], which reported good agreement between experimental measurements, three-dimensional numerical predictions and established natural-convection correlations for an aluminium plate-fin heat sink. Although differences in geometry and operating conditions preclude direct point-by-point validation of the present TPMS architectures, this experimental evidence provides an independent benchmark for the underlying buoyancy-driven conjugate heat-transfer methodology. Accordingly, the combined mesh-sensitivity assessment, analytical radiation verification, energy-balance check, flow-regime verification and comparison with published experimental literature provide confidence in the predicted comparative thermal trends. Direct experimental validation of the five DMLS-manufactured AlSi10Mg architectures remains an important subject for future work.

## 4. Discussion

The present study demonstrates that the thermal performance of DMLS-compatible passive heat sinks is governed by the interaction between geometric topology, natural-convection flow, and thermal radiation rather than by surface area alone. Previous studies have consistently reported that increasing heat-transfer area improves passive cooling by enhancing convection [2,3,4,27]; however, the present results indicate that this relationship becomes increasingly complex for lattice and triply periodic minimal surface (TPMS) architectures. As architectural complexity increases, the additional internal surface area may not be fully utilised because restricted buoyancy-driven airflow reduces convective effectiveness. Therefore, the optimisation of passive heat sinks requires balancing heat-transfer area with airflow permeability rather than maximising surface area alone [1,2,6].

The explicit inclusion of thermal radiation is particularly relevant to the investigated AM architectures because their large exposed and geometrically complex surfaces can substantially increase radiative heat exchange, making radiation an important component of overall passive heat dissipation [2,15,16]. For fin-based heat sinks, effective thermal performance requires sufficient surface area while maintaining buoyancy-driven airflow. In contrast, lattice and TPMS architectures introduce greater surface-area density but also increased flow resistance and complex internal transport pathways. Consequently, increased surface area alone does not guarantee superior passive cooling; performance depends on the balance between thermal spreading, airflow accessibility, topology, permeability and radiative heat exchange. The continuous and smoothly curved gyroid topology provides a favourable combination of these characteristics compared with the more discrete Schwarz P and BCC diamond networks [21,22,23].

Among the investigated geometries, the gyroid lattice delivered the lowest average operating temperature and combined thermal resistance, confirming the advantages of continuously interconnected TPMS structures for passive thermal management. Unlike the Schwarz P and BCC diamond lattices, whose node-based cellular networks restricted internal airflow, the gyroid provided smoother flow pathways that promoted more effective thermal spreading throughout the structure. These observations are consistent with recent investigations of TPMS heat exchangers and heat sinks, which have shown that continuously curved minimal surfaces improve fluid transport while maintaining a high surface-area-to-volume ratio [38,39]. Quantitatively, the gyroid architecture achieved a combined thermal resistance of 3.30 °C W^−1^, corresponding to reductions of approximately 12.1%, 15.2%, 17.3% and 17.7% relative to the perforated planar-fin, curved radial-fin, Schwarz P and BCC diamond heat sinks, respectively. The observed performance hierarchy is consistent with previous TPMS heat-transfer studies. Baobaid et al. [37] demonstrated the potential of porous TPMS architectures to enhance free-convection heat transfer, while Tang et al. [39] showed that diamond, gyroid and related TPMS topologies exhibit substantially different convective behaviour because of differences in surface morphology and internal transport pathways. Similarly, studies of gyroid-based electronic cooling [19] and TPMS heat-transfer systems [38,39,40] demonstrate that continuously interconnected architectures can enhance thermal transport by combining high surface-area density with favourable fluid pathways. These studies support the present finding that topology and permeability, rather than surface area alone, govern the thermal effectiveness of TPMS heat sinks. Direct comparison of absolute thermal-resistance values between studies should, however, be treated carefully because heat-sink dimensions, heat input, cooling regime, surface emissivity, material properties and definitions of thermal resistance vary considerably among published investigations [10,38,39,40].

An important outcome of this work is the significant contribution of thermal radiation to passive cooling. Radiation accounted for approximately 46–73% of the total heat dissipation, demonstrating that radiative exchange represents a substantial component of passive cooling under the investigated conditions. The increasing radiative contribution observed with increasing architectural complexity suggests that radiation becomes progressively more important as the exposed surface area increases and the operating temperature remains sufficiently high. These findings emphasise that coupled convective-radiative analyses are essential for accurately assessing metallic heat sinks manufactured by additive manufacturing, particularly for AlSi10Mg components whose complex geometries provide extensive radiating surfaces [13,19,41]. From a manufacturing perspective, the results further demonstrate that thermal optimisation must be considered alongside additive manufacturing constraints. Although computational geometry optimisation can generate highly efficient thermal structures, many designs remain impractical because of unsupported overhangs, enclosed cavities, or feature sizes below DMLS manufacturing limits. By evaluating representative DMLS-compatible geometries, the present study provides a more realistic assessment of passive heat-sink performance that is directly applicable to engineering practice. This approach bridges the gap between computational optimisation and manufacturable thermal-management solutions [9,19,41].

Numerical reliability was established through mesh-sensitivity analysis, domain sensitivity, Stefan–Boltzmann verification, energy conservation and flow-regime assessment, supported by published experimental–numerical natural-convection data. Direct experimental validation of DMLS AlSi10Mg prototypes remains future work [36].

Future work should quantify the influence of manufacturing-induced surface roughness, emissivity modification, build orientation, alternative heat loads and forced-convection conditions, together with structural stiffness, fabrication time and broader multi-objective manufacturability considerations [33,40].

## 5. Conclusions

This study demonstrated that the thermal performance of DMLS-compatible AlSi10Mg passive heat sinks is governed by the coupled interaction of geometry, conductive heat spreading, buoyancy-driven natural convection, and thermal radiation. The results confirm that increased surface area alone does not ensure improved passive cooling because its effectiveness depends strongly on airflow accessibility and utilisation of the available heat-transfer surface.

The principal findings from this study were:

- The gyroid exhibited the best overall thermal performance, achieving the lowest average surface temperature of 82.02 °C and combined thermal resistance of 3.30 °C W^−1^, corresponding to reductions of approximately 12.1–17.7% relative to the other investigated architectures.
- Topology was more influential than surface area alone. The continuous gyroid architecture provided favourable thermal spreading and internal transport pathways, whereas the more restrictive Schwarz P and BCC diamond structures reduced effective utilisation of their available surface area.
- Thermal radiation was a major heat-transfer mechanism, accounting for approximately 46–73% of total heat dissipation. This demonstrates the importance of coupled convective–radiative modelling when evaluating passive AM heat sinks with extensive exposed surfaces.
- Manufacturability was incorporated directly into the design framework. Restricting the investigation to DMLS-compatible AlSi10Mg architectures enabled thermal performance to be assessed alongside practical LPBF geometric constraints.
- Numerical reliability was assessed through mesh-sensitivity analysis, Stefan–Boltzmann analytical verification, energy-conservation checks and confirmation of the applicable laminar natural-convection regime.

Overall, the gyroid provided the most favourable balance between heat-transfer surface utilisation, thermal spreading, airflow accessibility, and radiative heat exchange, demonstrating the potential of TPMS architectures for DMLS-enabled passive thermal management. Experimental validation of manufactured AlSi10Mg prototypes is recommended to quantify the effects of surface roughness, emissivity variation, and manufacturing tolerances.

## Figures and Tables

**Table 1:** Mesh-sensitivity assessment for Heat Sink 1.

Mesh Refinement	T_MAX_ (°C)	Change from Previous (%)
3	92.93	--
4	92.56	0.40%
5	91.88	0.74%
6	91.37	0.55%

**Table 2:** Maximum, minimum and average temperatures with temperature uniformity index for the five heat sinks.

Component	*Tmax* (°C)	*Tmin* (°C)	*Tavg* (°C)	UI
Heat Sink 1	92.56	87.16	89.36	0.060
Heat Sink 2	93.51	90.09	91.39	0.037
Heat Sink 3	95.85	91.30	92.83	0.049
Heat Sink 4	95.58	92.32	93.30	0.035
Heat Sink 5	84.17	78.23	82.02	0.072

**Table 3:** Simulated average heat flux for the five heat-sink architectures.

Component	$q^{\prime \prime }(W/m^{2})$
Heat Sink 1	449.94
Heat Sink 2	472.01
Heat Sink 3	487.00
Heat Sink 4	490.42
Heat Sink 5	393.39

**Table 4:** Comparison of the simulated convective and radiative heat-transfer contributions for the five passive heat-sink architectures under a constant heat input of 18 W.

Component	$Q_{in}(W)$	$Q_{rad}(W)$	$Q_{conv}(W)$	ΣQ(W)	Radiation (%)	Convection (%)
Heat Sink 1	18	8.34	8.81	17.15	46.33	48.94
Heat Sink 2	18	10.19	6.84	17.03	56.61	38.00
Heat Sink 3	18	11.34	5.70	17.04	63.01	31.69
Heat Sink 4	18	10.77	6.24	17.01	59.84	34.67
Heat Sink 5	18	13.05	4.21	17.27	72.52	23.43

**Table 5:** Surface area together with the extracted convective and radiative heat-transfer coefficients for the five passive heat-sink architectures.

Component	A (m^2^)	T_avg_ (°C)	ΔT (°C)	Q_conv_ (W)	Q_rad_ (W)	h_conv_ (W/m^2^K)	h_rad_ (W/m^2^K)
Heat Sink 1	0.0184	89.36	64.36	8.81	8.34	7.43	7.03
Heat Sink 2	0.0216	91.39	66.39	6.84	10.19	4.77	7.10
Heat Sink 3	0.0232	92.83	67.83	5.70	11.34	3.62	7.19
Heat Sink 4	0.0214	93.30	68.30	6.24	10.77	4.26	7.36
Heat Sink 5	0.0330	82.02	57.02	4.21	13.05	2.24	6.93

**Table 6:** Convective and combined thermal resistance values.

Component	(h_conv_ + h_rad_) (W/m^2^K)	R_conv_ (°C/W)	R_sink_ (°C/W)
Heat Sink 1	14.47	7.30	3.75
Heat Sink 2	11.88	9.69	3.89
Heat Sink 3	10.81	11.90	3.99
Heat Sink 4	11.63	10.94	4.01
Heat Sink 5	9.17	13.52	3.30

**Table 7:** Estimated mass and material cost of each heat-sink design.

Component	Mass (G)	Cost (£)
Heat Sink 1	49.44	£3.96
Heat Sink 2	61.01	£4.88
Heat Sink 3	60.06	£4.81
Heat Sink 4	80.60	£6.45
Heat Sink 5	70.61	£5.65

**Table 8:** Comparison of simulated and analytical radiative heat-transfer rates.

Component	Q_rad_/Simulation (W)	Q_rad_/Calculated (W)	% Error
Heat Sink 1	8.34	8.69	4.29%
Heat Sink 2	10.19	10.63	4.34%
Heat Sink 3	11.34	11.74	3.53%
Heat Sink 4	10.77	10.93	1.48%
Heat Sink 5	13.05	13.34	2.20%

## Data Availability

The original contributions presented in this study are included in the article. Further inquiries can be directed to the corresponding author.

## Nomenclature

Symbol/Acronym	Definition	Unit
A	Heat-transfer surface area	m^2^
h	Convective heat-transfer coefficient	W m^−2^ K^−1^
Nu	Nusselt number	–
Q	Heat-transfer rate	W
Ra	Rayleigh number	–
Rth	Thermal resistance	°C W^−1^
T	Temperature	°C or K
ε	Surface emissivity	–
σ	Stefan–Boltzmann constant	W m^−2^ K^−4^
AM	Additive manufacturing	–
BCC	Body-centred cubic	–
CAD	Computer-aided design	–
CFD	Computational fluid dynamics	–
DfAM	Design for Additive Manufacturing	–
DMLS	Direct Metal Laser Sintering	–
LPBF	Laser Powder Bed Fusion	–
SWFS	SolidWorks Flow Simulation	–
TPMS	Triply periodic minimal surface	–
UI	Temperature uniformity index	–
